# Endophytic bacteria *Klebsiella* spp. and *Bacillus* spp*.* from *Alternanthera philoxeroides* in Madiwala Lake exhibit additive plant growth-promoting and biocontrol activities

**DOI:** 10.1186/s43141-023-00620-8

**Published:** 2023-11-30

**Authors:** Soma Biswas, Indhu Philip, Saranya Jayaram, Suma Sarojini

**Affiliations:** Department of Life Sciences, CHRIST (Deemed to Be University), Bangalore-29, India

**Keywords:** Sustainability, Halotolerant, Bacterial endophyte, Biofertilizer, Antimicrobial, IAA, RSM, Bioinoculant, *V*. *unguiculata*

## Abstract

**Background:**

The worldwide increase in human population and environmental damage has put immense pressure on the overall global crop production making it inadequate to feed the entire population. Therefore, the need for sustainable and environment-friendly practices to enhance agricultural productivity is a pressing priority. Endophytic bacteria with plant growth-promoting ability and biocontrol activity can strongly enhance plant growth under changing environmental biotic and abiotic conditions. Herein, we isolated halotolerant endophytic bacteria from an aquatic plant, *Alternanthera philoxeroides*, from the polluted waters of Madiwala Lake in Bangalore and studied their plant growth promotion (PGP) and biocontrol ability for use as bioinoculant.

**Results:**

The isolated bacterial endophytes were screened for salt tolerance ranging from 5 to 15% NaCl concentration. *Klebsiella pneumoniae* showed halotolerant up to 10% NaCl and *Bacillus amyloliquefaciens* and *Bacillus subtilis* showed up to 15%. All three strains demonstrated good PGP abilities such as aminocyclopropane-1-carboxylic acid (ACC) deaminase activity, phosphate solubilization, ammonia production, and nitrogen fixation. In addition, *K*. *pneumoniae* also exhibited high indoleacetic acid (IAA) production (195.66 ± 2.51 µg/ml) and potassium solubilization (2.13 ± 0.07 ppm). *B*. *amyloliquefaciens* and* B*. *subtilis* showed good extracellular enzyme production against cellulase, lipase, protease, and amylase. Both the isolates showed a broad spectrum of antimicrobial activity against the tested organisms. The optimization of IAA production by *K*. *pneumoniae* was done by the response surface methodology (RSM) tool. Characterization of IAA produced by the isolate was done by gas chromatography-mass spectrometry (GCMS) analysis. The enhanced plant growth-promoting ability of *K*. *pneumoniae* was also demonstrated using various growth parameters in a pot trial experiment using the seeds of *Vigna unguiculata*.

**Conclusion:**

The isolated bacterial endophytes reported in this study can be utilized as PGP promotion and biocontrol agents in agricultural applications, to enhance crop yield under salinity stress. The isolate *K*. *pneumoniae* may be used as a biofertilizer in sustainable agriculture and more work can be done to optimize the best formulations for its application as a microbial inoculant for crops.

**Supplementary Information:**

The online version contains supplementary material available at 10.1186/s43141-023-00620-8.

## Background

With the increasing trend in the world’s population, there is a looming threat of inadequacy in food supply to serve the entire population. Researchers all around the world are trying to fix this problem by developing sustainable ways of increasing agricultural productivity. The most important sustainable way to increase agricultural yield is by the use of microorganisms instead of chemical fertilizers [1]. In nature, plant tissues are colonized by many types of microbes such as bacteria, fungi, algae, archaea, and amoebae. These endophytic microbes live inside the plant tissue without causing any symptoms of disease thereby, maintaining a complementary relationship with the host plant [2]. Endophytes confer several ecological benefits to the host plant, like increased stress tolerance, PGP, and resistance against pathogens [3, 4].

In recent times, the science of endophyte biology has gained much attention due to its potential to produce novel bioactive compounds with anticancer, antimicrobial, antioxidant, immunosuppressive, PGP, and insecticidal properties, with pharmaceutical and commercial importance [5]. Plant growth-promoting endophytic bacteria (PGPEB) assist in plant growth both directly and indirectly through various mechanisms. The direct mechanism involves synthesizing phytohormones and fixing nitrogen, by enhancing the solubilization of minerals like phosphate, zinc, potassium, and 1-aminocyclopropane-1-carboxylic acid (ACC) deaminase activity, all of which can have an effect either singly or cumulatively on plant growth. The most important plant hormones produced by endophytic bacteria are auxins, cytokinins, gibberellins, abscisic acid, ethylene, etc. The indoleacetic acid (IAA) compound synthesized by endophytic bacteria has a direct impact on various plant physiological processes. This includes promoting growth by cell elongation and differentiation, stimulating the formation of lateral roots, and influencing chlorophyll pigmentation [6, 7]. The common producers of IAA belong to the bacterial genera *Pseudomonas*, *Klebsiella*, *Azospirillum*, *Azotobacter*, *Anterobacter*, *Rhizobium*, *Bacillus*, *Rhodococcus*, and *Pantoea* [8]. The presence of ACC deaminase enzyme in endophytic bacteria is a pivotal factor in promoting plant growth even under abiotic stress by reducing the ethylene levels in the host plant [9]. PGPEB possesses the capacity to generate organic acids, enzymes, antibacterial substances, cyanide production, trigger systemic defense mechanisms, and generate siderophores, collectively contributing to the indirect enhancement of plant growth [7]. They also protect the plant from phytopathogens, suppressing plant diseases via ammonia and antibiotic production, modifying the production of stress-related proteins, and by phytoremediation (hydrocarbon and heavy metal removal), thereby acting as biocontrol agents [6]. They protect plants from various environmental stresses like salinity, drought, and temperature, thereby enhancing their capability to acclimatize to stressful conditions [10]. Halo-tolerant PGPEB has been reported to help plants tolerate high salinity through various mechanisms—by producing reactive oxygen species (ROS) scavengers, by reducing osmotic, ion, and oxidative stress, by producing ACC-deaminase enzymes to breakdown ethylene precursor ACC [11], and by the modulation of phytohormones, siderophores, and exopolysaccharides [12–14].

The plant chosen for this study *Alternanthera philoxeroides* (alligator weed) is an interesting one to study the endophytic microbial flora affecting plant growth. Its abundance in and around the Madiwala Lake, Bangalore, made it an option of choice for the proposed study [15]. Research shows that stressful conditions have promoted greater tolerance to herbivory in *A*. *philoxeroides* [16]. Leitner et al. (2019) have isolated a rhizospheric bacteria, *Exiguobacterium acetylicum* with leachate degradation potential, from *A*. *philoxeroides* (Mart) Griseb [17]. Studies have revealed the isolation, culture, and identification of various endophytic fungi from *A*. *philoxeroides* [18]. However, very few studies have focused on isolating endophytic bacteria with potential applications from this plant. To the best of our knowledge, this is the first report of three endophytic bacteria *K*. *pneumoniae*, *B*. *amyloliquefaciens*, and* B*. *subtilis* isolated from *A*. *philoxeroides* with PGP potential and biocontrol activity, which can be exploited to develop bioinoculants for sustainable and environment friendly agricultural applications.

## Methods

### Chemicals and reagents

Ammonium molybdate, stannous acid, calcium carbonate, gelatine, tryptophan, and peptone were brought from HIMEDIA (India). All reagents such as Kovac’s, Salkowski, Nessler’s, Chlorostannous acid, Chloromolybdic acid, and HPLC-grade solvents were purchased from HIMEDIA (India). Other chemicals, antibiotics, and reagents used in this study were obtained from HIMEDIA (India). All chemicals and reagents used in this study were of analytical grade.

### Isolation of endophytic bacteria

For the isolation of endophytes, the *A*. *philoxeroides* plant was collected from Madiwala Lake, Bangalore, India (Lat N 12° 54′ 3.492′′ Long E 77° 37′ 4.0044′′). The isolation was carried as per the modified method described by Panigrahi et al. (2018) [19]. The plant sample was first cleaned extensively in running tap water for 15 min to remove dirt and debris, then washed with Tween 20 detergent solution for 2 min, and later with distilled water for three times. The sample was then surface sterilized by dipping in 70% ethanol for 2 min, followed by rinsing with sterile distilled water for seven times. The surface sterilized plant sample was then blot-dried using sterile filter paper. For sterility check, 0.1 ml of the last washed sterile distilled water was spread plated on Luria–Bertani (LB) agar plate. Surface sterilized plant samples of leaf, stem, and root were cut into small parts of size 1–3 mm. The stem and root parts were cut from the middle and placed inverted onto an LB agar plate. Leaf samples were cut from the margin and from the midrib region and placed onto an LB agar plate. The plates were kept for incubation at 37 °C for 1 week. The plates were also checked intermittently for bacterial growth. Colonies of bacterial endophytes with different morphology were selected for further studies. To obtain pure colonies, isolated bacterial endophytes were streaked on nutrient agar plates. The pure cultures were streaked on nutrient agar slants and kept at 4 °C for further use.

### Phenotypic and biochemical characterization

Bacterial endophytes were observed carefully to characterize the morphology. Colony characters that were observed included size, pigmentation, form, margin, elevation, and texture. Biochemical tests such as Indole, Methyl red, Voges-Proskauer, Citrate, Urease, Triple Sugar Iron, and Gelatinases and Gram staining procedures were performed for the isolates.

### Molecular identification

Genomic DNA samples were isolated from the selected bacterial isolates and were used as templates for polymerase chain reaction (PCR) using 16S rRNA-F and 16S rRNA-R primers. The Universal 16S rRNA bacterial primer 27F (5′-AGAGTTTGATCCTGGCTCAG-3′) and 1392R (5′-GGTTACCTTGTTACGACTT-3′) were used to amplify the gene. The total PCR reaction mixture was 50 μl, comprising 50 ng genomic DNA, 50 mM each of forward and reverse primers, 200 mM dNTPs, 1X PCR assay buffer, and 2 U Taq polymerase. Amplification was carried out for 35 cycles in a Thermal cycler with conditions involving an initial denaturation for 3 min at 94 °C, followed by cyclic denaturation for 30 s at 94 °C, annealing for 1 min at 58 °C, elongation for 2 min at 72 °C, and final extension for 7 min at °C. The PCR product formation was checked by 1% agarose gel electrophoresis, purified, and further subjected to sequencing [20]. DNA sequencing was performed with forward and reverse primers, 27F, and 1392R, using BDT v3.1 Cycle sequencing kit on ABI 3730xl Genetic Analyzer. The 16S rRNA gene sequence was used to carry out BLAST with the ‘nr’ database of the NCBI GenBank database. Based on the maximum identity score, the first ten sequences were selected and aligned using the multiple alignment software program Clustal W. Distance matrix, and a phylogenetic tree was constructed using MEGA 10 [21, 22].

### Salt tolerance

The isolates were tested for their ability to tolerate salinity by inoculating on nutrient agar (NA) media having different NaCl concentrations ranging from 5 to 15% and incubated at 37 °C for 24–72 h [23].

### Determination of plant growth-promoting attributes of bacterial endophytes

Several qualitative and quantitative tests were performed to determine important PGP attributes of the isolates, which include extracellular enzyme activity, IAA production, ammonia production, nitrogen fixation, ACC deaminase activity, phosphate solubilization, and potassium solubilization.

### Extracellular enzyme production

Bacterial isolates were screened for the secretion of different extracellular enzymes namely cellulase, amylase, lipase, and protease. After 48 h of incubation enzymatic index (EI) was calculated [24].

Enzymatic index (EI) = (Colony diameter (cm) + Halozone diameter (cm)) / Colony diameter (cm).

### Cellulase production

Cellulose degrading ability was determined by inoculating the isolates in cellulose Congo red media consisting of MgSO_4_.7H_2_O 0.25 g/L, KH_2_PO_4_ 0.5 g/L, cellulose 2 g/L, gelatin 2 g/L, Congo red 0.2 g/L, and agar 15 g/L. Plates were kept for incubation at 37 °C for 48 h. The formation of a clear zone around and beneath the colony indicated enzymatic degradation of cellulose [25].

### Amylase production

For amylase production, bacterial isolates were inoculated onto Mineral salt media (MSM) consisting of NaNO_3_ 5 g/L, MgSO_4._7H_2_O 0.5 g/L, KH_2_PO_4_ 1 g/L, K_2_HPO_4_ 2 g/L, KCl 0.1 g/L, FeSO_4_.7H_2_O 0.02 g/L, CaCl_2_ 0.01 g/L, agar 15 g/L, and incubated at 37 °C for 48 h. After incubation, the plates were immersed in a 1% iodine solution. The appearance of a clear zone around the colony indicated the activity of amylase enzyme [26].

### Protease production

Production of protease enzymes by the isolates was carried out using skim milk agar media consisting of yeast extract 2.5 g/l, tryptone 5 g/l, glucose 1 g/l, agar 18 g/l, and skim milk 5%. The isolates were spot inoculated and incubated at 37 °C for 24–48 h. Plates were further checked for the appearance of a clear zone around the colony indicating the proteolytic ability of the isolates [27].

### Lipase production

Production of lipase was performed in media containing peptone 5 g/L, yeast extract 3 g/L, tributyrin oil 4 ml/L (v/v), and agar 18 g/L. Isolates were inoculated onto the media and incubated at 37 °C for 24–48 h. The advent of clearing zones around the colony indicated the lipolytic ability of the bacterial isolates [28].

### IAA production

The potentiality of the isolated bacterial endophytes to produce IAA was determined by inoculating the isolate in 5 ml of nutrient broth with 1% tryptophan and incubating for 72 h in a shaker at 37 °C and 120 rpm. The culture was centrifuged at 10,000 rpm for 15 min and the supernatant was collected. Further, 2 ml of Salkowski’s reagent was added to 1 ml of the supernatant and incubated at room temperature for 30 min under dark conditions. The appearance of pink to red indicated the presence of IAA. Quantitative estimation of IAA production was performed using a UV spectrophotometer based on the method as described by Gordon and Weber [29]. All experiments were performed in triplicates.

### Ammonia production

The endophytic isolates were inoculated in 5 ml of peptone water media consisting of peptone 20 g/l and sodium chloride 5 g/l and incubated at 37 °C for 4 days. The culture was then centrifuged at 10,000 rpm for 10 min and the supernatant was collected. Further, to 1 ml of culture supernatant, 1 ml of Nessler’s reagent was added. The formation of a dark yellow to brown color indicated the presence of ammonia production. Quantitative estimation of ammonia production was done using a UV spectrophotometer. All experiments were performed in triplicates [23].

#### Nitrogen fixation, ACC deaminase activity, and HCN (hydrogen cyanide) production

The isolated bacterial endophytes were further screened for their nitrogen-fixing ability by culturing them on Jensen’s media consisting of sucrose 20 g/L, NaCl 0.5 g/L, K_2_HPO_4_ 1 g/L, FeSO_4_.7H_2_O 0.1 g/L, NaMoO_4_ 0.005 g/L, MgSO_4._7H_2_O 0.5 g/L, CaCO_3_ 2 g/L, agar 15 g/L. The cultures were inoculated on Jensen’s media and incubated at 37 °C for 3–4 days. The presence of growth of bacterial colonies confirms the nitrogen-fixing capability of the isolates [30, 31].

The ACC deaminase activity was determined by inoculating the isolates on Dowrkin-Foster (DF) salts minimal medium supplemented with either ammonium sulfate or ACC, and incubated at 37 °C for 4 days. The media consists of Na_2_HPO_4_ 6 g/L, MgSO_4_.7H_2_O 0.2 g/L, KH_2_PO_4_ 4 g/L, FeSO_4_.7H_2_O 1 mg/L, MnSO_4_ 10 μg/L, ZnSO_4_ 70 μg/L, H_3_BO_3_ 10 μg/L, CuSO_4_ 50 μg/L, gluconic acid 2 g/L, MoO_3_ 10 μg/L, citric acid 2 g/L, glucose 2 g/L, ammonium sulfate 2 g/L, and agar 12 g/L. The presence of growth on culture media is an indication of the presence of ACC deaminase enzyme activity [32, 33].

For hydrogen cyanide (HCN) production, the bacterial isolates were streaked on King’s B medium supplemented with 0.4% (w/v) glycine. A Whatman filter paper was kept soaked in alkaline picric acid solution (0.5% picric acid in 2% Na_2_CO_3_) for 30 min and placed on the upper lids of Petri plates and kept for incubation at room temperature for 4 days. The change of yellow color filter paper to red-brown color is indicative of HCN production [34].

### Phosphate solubilization

Qualitative estimation of phosphate solubilization by bacterial isolates was performed using Pikovskaya’s agar media consisting of Ca_3_(PO_4_)_2_ 5 g/L, (NH_4_)_2_SO_4_ 0.5 g/L, KCl 0.2 g/L, MgSO_4_.7H_2_O 0.1 g/L, FeSO_4_.7H_2_O 0.002 g/L, NaCl 0.2 g/L, glucose 10 g/L, yeast extract 0.5 g/L, MnSO_4_.2H_2_O 0.002 g/L, agar 20 g/L with 500 µl of 0.5% bromocresol green indicator. The isolates were inoculated onto the media and incubated at 37 °C for 48 h to check the appearance of a yellow zone around the colony indicative of a positive result. The phosphate solubilization index (PSI) was also measured [35, 36].

### Phosphate solubilization index = (colony diameter+halo zone) / colony diameter

Quantitative estimation of phosphate was performed by inoculating the bacterial isolates in Pikovskaya broth and incubated at 37 °C for 4 days. The broth culture was then centrifuged; and to 1 ml of supernatant 10 ml of chloromolybdic acid (15 g of ammonium molybdate dissolved in 400 ml of warm distilled water was added, to this add 342 ml of 12 N HCl was added to make up the volume to 1000 ml) was added. It was shaken well, diluted to 45 ml, and then 0.25 ml of chlorostannous acid (2.5 g of stannous acid SnCl_2_.2H_2_O was added, and dissolved in 10 ml of conc. HCl and made up the volume to 100 ml with distilled water) was added. The optical density of the blue-colored solution was measured at 600 nm using a UV spectrophotometer. All experiments were performed in triplicates. A standard curve of KH_2_PO_4_ (10–100 µg/ml) was prepared [37, 38].

### Potassium solubilization

The isolates were screened for potassium solubilization qualitatively by inoculating onto Aleksandrow agar media consisting of glucose 5 g/L, MgSO_4_.7H_2_O 0.005 g/L, FeCl_3_ 0.1 g/L, CaCO_3_ 2 g/L, CaPO_4_ 2 g/L, agar 20 g/L, mica 3 g/L, 500 µl of 0.5% bromocresol green, and incubating at 37 °C for 72 h. The presence of the halo zone around the colony is indicative of positive results [39].

Quantitative estimation was done for the isolates showing a clear zone in the Aleksandrow agar medium. The isolates were inoculated in 50 ml of Aleksandrow broth (pH 7) containing mica (3 g/L) as a potassium source. Then, 10 µl of bacterial isolate (10^6^ CFU/ml) was inoculated onto the autoclaved Aleksandrow broth and incubated at 30 °C and 120 rpm for 9 days. The culture samples were taken out at different incubation times (3rd, 6th, and 9th day) to determine the amount of potassium solubilized. The culture obtained was centrifuged at 10,000 rpm for 10 min at 4 °C and the cell-free supernatant was further used for determining available potassium using atomic absorption spectrophotometer (AAS). All experiments were performed in triplicates [40].

### Antimicrobial activity assay

Antibacterial activity was assayed by using the agar well diffusion method against six test organisms—gram-positive *Bacillus cereus*, *Streptococcus pneumoniae*, and gram-negative *Escherichia coli*, *Vibrio harveyi*, *Vibrio parahaemolyticus*, and *Vibrio vulnificus*. Further, 0.1 ml of overnight grown test bacterial suspension were spread plated into nutrient agar plates using a sterile L-shaped cell spreader. Wells were made on the plates using a cork borer of 5 mm diameter. Then, 50 µl of the broth culture of bacterial isolates was poured into the well. Plates were subsequently incubated at 37 °C for 24 h and the zone of inhibition was measured [41].

Antifungal activity was investigated in vitro against three fungi—*Fusarium solani*, *Talaromyces amestolkiae*, and *Cladosporium tenuissimum*—by dual culture technique. The fungal cultures were grown on Sabouraud Dextrose Agar (SDA) for 5 days and fungal discs of 5 mm diameter were cut and placed onto another SDA plate. Further, the overnight grown endophytic bacterial culture in NA was streaked on four corners of the plate and kept for incubation at 28 ± 2 °C for 3 days. For the control plate, only a fungal disc was placed at the center [42]. Finally, the antifungal activity was confirmed by the zone of inhibition, and percent inhibition was calculated according to the following formula [43].

$$\mathrm{Percent\,Inhibition }\,\left(\%\right)={\mathrm{F}}_{\mathrm{fed}}-{\mathrm{T}}_{\mathrm{fed}}/{\mathrm{F}}_{\mathrm{fcd}}- {\mathrm{F}}_{0} \times 100$$

where F_fcd_ refers to fungal colony diameter on the control SDA plate, T_fcd_ is the fungal colony diameter on the test SDA plate, F_0_ is the test fungal disc diameter (5 mm) on the SDA plates.

### Characterization of IAA production using GCMS

For the purification of IAA, 100 ml of nutrient broth supplemented with 1% of tryptophan was inoculated with *K*. *pneumoniae* MEBAphS1 and incubated at 37 °C in shaking condition at 120 rpm for 10 days. The culture was further centrifuged at 10,000 rpm for 10 min and the supernatant was collected. The culture supernatant was acidified to pH 3 by adding 6 N HCl and extracted three times with an equal volume of ethyl acetate. The organic phase was then collected and evaporated in a rotary evaporator to obtain IAA, which was reconstituted with 3 ml of ethyl acetate and stored at 4 °C for further analysis [19, 44].

The purified IAA was then analyzed using gas chromatography-mass spectrometry (GCMS). Clarus 680 GC was used for the analysis, which was employed with a fused silica column, packed with Elite-5MS (5% biphenyl 95% dimethylpolysiloxane, 30 m × 0.25 mm ID × 250 μm df). The components were separated using Helium as carrier gas at a constant flow of 1 ml/min. The injector temperature was kept at 260 °C during the chromatographic run. An extract sample of 1 μL was injected into the instrument at the oven temperature as follows: 60 °C (2 min); followed by 300 °C at the rate of 10 °C min^−1^ and 300 °C, where it was held for 6 min. The mass detector conditions were set as: transfer line temperature of 240 °C; ion source temperature of 240 °C; ionization mode electron impact at 70 eV, a scan time of 0.2 s, and scan interval of 0.1 s. Fragment sizes ranging from 40 to 600 Da were detected. The spectra of the components were compared with the database of the spectrum of known components stored in the GCMS NIST (2008) library.

### Optimization of IAA production using response surface methodology

In order to determine the optimum conditions for maximizing IAA production from the isolate *K*. *pneumoniae*, a statistical tool, response surface methodology (RSM), was employed. The central composite design (CCD), which is a part of response surface methodology, was used to evaluate the interaction between different factors and to obtain optimal response. The experimental design was formulated based on the three selected independent variables such as pH, incubation time, and tryptophan concentration (Table 1). A quadratic model in Eq. (1) has been used to express the response as a function of the three independent variables [45].

**Table 1:** Experimental range and level used in the optimisation of IAA production by endophyte *Klebsiella pneumoniae* MEBAphS1 from *Alternanthera philoxeroides* using CCD

Independent Variables	Symbols	Range and Level	
		Level 1	Level 2
Incubation time (days)	A	0	20
pH	B	4	9
Tryptophan Concentration (%)	C	0.1	2

1 $$\mathrm Y={\mathrm b}_0+{\mathrm b}_1\mathrm A+{\mathrm b}_2\mathrm B+{\mathrm b}_3\mathrm C+{\mathrm b}_4\mathrm{AB}+{\mathrm b}_5\mathrm{AC}+{\mathrm b}_6\mathrm{BC}+{\mathrm b}_7\mathrm A^2+{\mathrm b}_8\mathrm B^2+{\mathrm b}_9\mathrm C^2$$

Where Y is IAA concentration (µg/ml), b_0_- b_9_ are the coefficients, A is incubation time (days), B is pH and C is tryptophan concentration (%).

### Pot trial experiments

A pot trial experiment was performed to evaluate the effect of endophytic bacteria *K*. *pneumoniae* on cowpea (*V*. *unguiculata*) plants. The seeds of cowpeas were purchased from the market and checked for their germination capacity prior to the start of the experiment. The seeds were first surface sterilized with 0.2% HgCl_2_ for 3–4 min followed by rinsing with sterile distilled water 12–15 times. The seeds were then kept soaked overnight in sterile water for germination. The germinated seeds were then kept soaked in 10^6^ cfu/ml of bacterial suspension cultured in nutrient broth with 0.2% tryptophan for 2 h. After that, the coated seeds were sowed at 2 cm depth onto the potting mixture of sterile soil and organic manure (1:1 ratio) on the pot tray. After 15 days, once the seedlings achieved a two-leaf stage, they were transplanted to medium-sized plastic pots (one plant per pot) and allowed to grow for another 15 more days. After 30 days, plants were harvested and fresh weight, dry weight, leaf number, root and shoot length, and chlorophyll content were determined. All experiments were carried out in pentaplicates. For checking the chlorophyll content in both *K*. *pneumoniae* (MEBAphS1) inoculated and control plants, fresh leaves were taken and macerated using 80% chilled acetone [46, 47]. The extract was then centrifuged and the OD of the supernatant was measured at 663 nm and 645 nm to quantify the pigment using the following formula:

$$\mathrm{Chlorophyll \,A }\left(\mathrm{mg}/\mathrm{ml}\right)= \left(12.7\times \mathrm{A}663-2.69\times \mathrm{A}645\right)\times \mathrm{final \,volume}$$

$$\mathrm{Chlorophyll \,B }\left(\mathrm{mg}/\mathrm{ml}\right)= \left(22.9\times \mathrm{A}645-4.68\times \mathrm{A}663\right)\times \mathrm{final \,volume}$$

### Statistical analysis

The results were analyzed statistically using IBM SPSS statistics 21 software and Microsoft Excel 2016 with at least three replicates and expressed as mean ± SD (standard deviation). One-way ANOVA was done to confirm the validity and variability of the results. One sample student’s *t* test was performed for comparison among the groups at *p* < 0.05.

## Results

### Isolation of bacterial endophytes

In this present study, nine bacterial endophytes were obtained by culturing the leaf, stem, and root of *A*. *philoxeroides*. The absence of bacterial growth in the control plate confirmed that the isolated microbes were indeed endophytes. The isolates were coded as MEBAphL1-L5 (from leaf), MEBAphS1-S2 (from stem), and MEBAphR1-R2 (from root). The morphological and biochemical test results for the potential isolates are listed in Tables 2 and 3.

**Table 2:** Colony characteristics of the selected bacterial endophytic strains from *Alternanthera philoxeroides*

Colony morphology	Bacterial Isolate		
	**MEBAphS1**	**MEBAphL4**	**MEBAphR1**
Size	Small	Moderate	Moderate
Pigmentation	No	No	Red
Form	Circular	Irregular	Irregular
Margin	Entire	Undulate	Curled
Elevation	Raised	Flat	Flat
Texture	Mucoid	Rough	Rough

**Table 3:** Biochemical characterization of selected bacterial endophytic strains from *Alternanthera philoxeroides*

Biochemical Tests	Bacterial isolate		
	**MEBAphS1**	**MEBAphL4**	**MEBAphR1**
Gram Staining	Gram negative	Gram positive	Gram positive
Shape	Rods	rods	rods
Indole	+	−	−
Methyl Red	−	−	−
Vogus-Proskauer	+	+	+
Citrate	+	−	−
Gelatinase	−	+	+
Urease	+	+	−
Triple sugar ion	Yellow butt/Yellow slant (lactose fermented)	Red butt/Yellow slant	Red butt/Yellow slant

### Identification of isolates

Based on phenotypic, biochemical characterization, and PGP properties, MEBAphS1, MEBAphL4, and MEBAphR1 were chosen for further studies. PCR done using 16S rRNA primers revealed a single 1500 bp band on a 1% agarose gel. Molecular identification of these isolates was done by 16S rRNA sequencing which revealed the species to be *K*. *pneumoniae* (MEBAphS1), *B*. *amyloliquefaciens* (MEBAphL4), and *B*. *subtilis* (MEBAphR1) respectively (Supplementary Fig. S1 A–C). These sequences were submitted to NCBI and the accession numbers were OM534607, ON259674, and ON259697 for *K*. *pneumoniae*, *B*. *amyloliquefaciens*, and *B*. *subtilis* respectively. The presence of these identified strains as endophytes of *A*. *philoxeroides* has not yet been reported.

### Salt tolerance

**Fig. 1:**
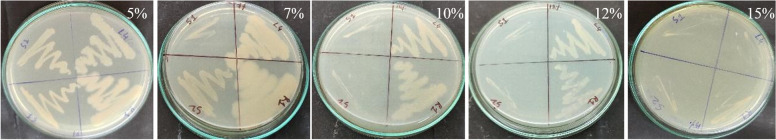
Growth of endophytic bacterial isolates from *Alternanthera philoxeroides* on nutrient agar media containing 5–15% NaCl concentration. S1 = MEBAphS1 (*Klebsiella pneumoniae*), L4 = MEBAphL4 (*Bacillus amyloliquefaciens)*, R1 = MEBAphR1 (*Bacillus subtilis*), S2 = MEBAphS2

The isolates were screened for salt tolerance at various salt concentrations (Fig. 1). The isolates *B*. *amyloliquefaciens* and *B*. *subtilis* have shown tolerance to 15% NaCl concentration while the isolate *K*. *pneumoniae* have shown tolerance to 10% NaCl concentration (Table 4). The salt-tolerant bacterial endophytes were further screened for their PGP and antimicrobial activity.

**Table 4:** Salt-tolerance level of isolated bacterial endophytes from *Alternanthera philoxeroides*

Endophyte isolate code	Endophyte species	Growth at different concentrations of NaCl				
		5%	7%	10%	12%	15%
MEBAphS1	*K. pneumoniae*	** + + + **	** + + **	** + **	**-**	**-**
MEBAphL4	*B. amyloliquefaciens*	** + + + **	** + + + **	** + + + **	** + + **	** + **
MEBAphR1	*B. subtilis*	** + + + **	** + + + **	** + + + **	** + + **	** + **

### Determination of plant growth-promoting activity

The isolates were screened for PGP properties by indirect mechanisms such as extracellular enzyme production (Table 5) and by direct mechanisms such as IAA, ammonia production, phosphate solubilization, nitrogen fixation, ACC deaminase, and potassium solubilization (Table 6).

**Table 5:** Enzymatic Index of the extracellular enzyme produced by the isolated endophytes from *Alternanthera philoxeroides*

Name of the isolate	Isolate identified as	Extracellular enzyme production (Enzymatic Index)			
		Cellulase	Lipase	Protease	Amylase
MEBAphS1	*K*. *pneumoniae*	–	–	–	2.13
MEBAphL4	*B*. *amyloliquefaciens*	3.72	2.11	2.15	2.08
MEBAphR1	*B*. *subtilis*	3.15	2.08	2.07	2.02

**Table 6:** Plant growth promotion properties of the isolated endophytic bacteria from *Alternanthera philoxeroides*

Bacterial Isolates	Plant Growth Promotion Properties										
	IAA (µg/ml)	Ammonia (µg/ml)	Phosphate Solubilization index	Phosphate solubilization (ppm)	Potassium solubilization (ppm)	Nitrogen fixation	ACC deaminase	HCN production	Antimicrobial activity		
									Gram-positive bacteria	Gram-negative bacteria	Fungus
*K. pneumoniae*	48.75 ± 3	2.92 ± 0.15	1.6	38.935 ± 0.31	2.13 ± 0.07	+ +	+ +	–	–	+ +	–
*B. amyloliquefaciens*	**–**	3.51 ± 0.15	1.4	30.708 ± 0.477	–	+ +	+ +	–	+ +	+ +	+ +
*B. subtilis*	**–**	3.37 ± 0.09	1.3	29.562 ± 0.312	–	+ +	+ +	–	+ +	+ +	+ +

### Extracellular enzyme production

Many endophytes are thought to possess extremozymes. The isolates were screened for extracellular enzyme production such as cellulase, amylase, lipase, and protease. The presence of a halo zone around the colonies was taken as positive results for cellulase, amylase, lipase, and protease enzymes. Isolate *K*. *pneumoniae* showed positive only for amylase enzyme while *B*. *amyloliquefaciens* and *B*. *subtilis* showed positive for all four enzymes. The enzymatic index was calculated for all the tested enzymes.

### IAA production

The isolates were evaluated for in vitro production of Indole acetic acid in nutrient broth media amended with 1% tryptophan as a precursor. Among the selected bacterial endophytes, MEBAphS1 showed good production of IAA as evidenced by the appearance of a reddish-pink color upon the addition of the Salkowski reagent. The IAA production was 48.75 ± 3 µg/ml for *K*. *pneumoniae*, *B*. *amyloliquefaciens*, and *B*. *subtilis* did not show IAA production. The strain *K*. *pneumoniae* identified in this study has good PGP ability in *A*. *philoxeroides* and its potential to enhance growth in other plants needs to be explored.

### Ammonia production

All the isolates tested for ammonia production showed positive results upon the addition of Nessler’s reagent by changing the color to yellowish brown. The intensity of color change was proportional to the amount of ammonia produced. Ammonia production was found to be 2.92 ± 0.15 µg/ml for *K*. *pneumoniae,* 3.51 ± 0.15 µg/ml for *B*. *amyloliquefaciens*, and 3.37 ± 0.09 µg/ml for *B*. *subtilis*.

### Nitrogen fixation, ACC deaminase activity, and HCN production

The isolates showed good growth in Jensen’s media after 48 h of incubation, thus, proving their nitrogen fixing ability. They were also found to be positive for ACC deaminase activity based on their ability to grow on DF salt minimal media supplemented with ACC or ammonium sulfate. For HCN production, the isolates showed negative results as the yellow picrate filter paper did not turn red-brown.

### Phosphate solubilization

**Fig. 2:**
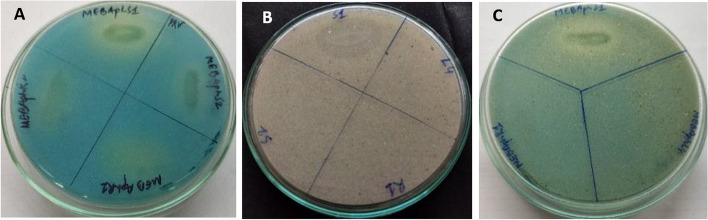
Screening of endophytic bacterial isolates from *Alternanthera philoxeroides* for **A** phosphate solubilization (in Pikovskaya media with 500 µl of 0.5% bromocresol green), **B** potassium solubilization (in Aleksandrow agar media), and **C** potassium solubilization (in Aleksandrow agar media with 500 µl of 0.5% bromocresol green)

The isolates were screened positive for phosphate solubilization in Pikovskaya’s media with bromocresol green indicator. The appearances of yellow halo zones around the colony were considered as positive results (Fig. 2A). The phosphate solubilization index (PSI) was 1.6, 1.4, and 1.3 for *K*. *pneumoniae*, *B*. *amyloliquefaciens*, and *B*. *subtilis* respectively. Quantitative estimation of phosphate solubilization by the isolates gave the following results—*K*. *pneumoniae*, *B*. *amyloliquefaciens*, and *B*. *subtilis* as 38.935 ± 0.31 ppm, 30.708 ± 0.477 ppm, and 29.562 ± 0.312 ppm, respectively.

### Potassium solubilization

The isolate *K*. *pneumoniae* showed positive for potassium solubilization by forming a halo zone around the colony when grown in Aleksandrow agar media with bromocresol green indicator after 48 h of incubation (Fig. 2B, C). The quantitative estimation of the isolate to solubilize potassium was found to be 2.13 ± 0.07 ppm after 192 h of incubation. *B*. *amyloliquefaciens* and *B*. *subtilis* isolates gave negative results for potassium solubilization.

### Antimicrobial activity

**Fig. 3:**
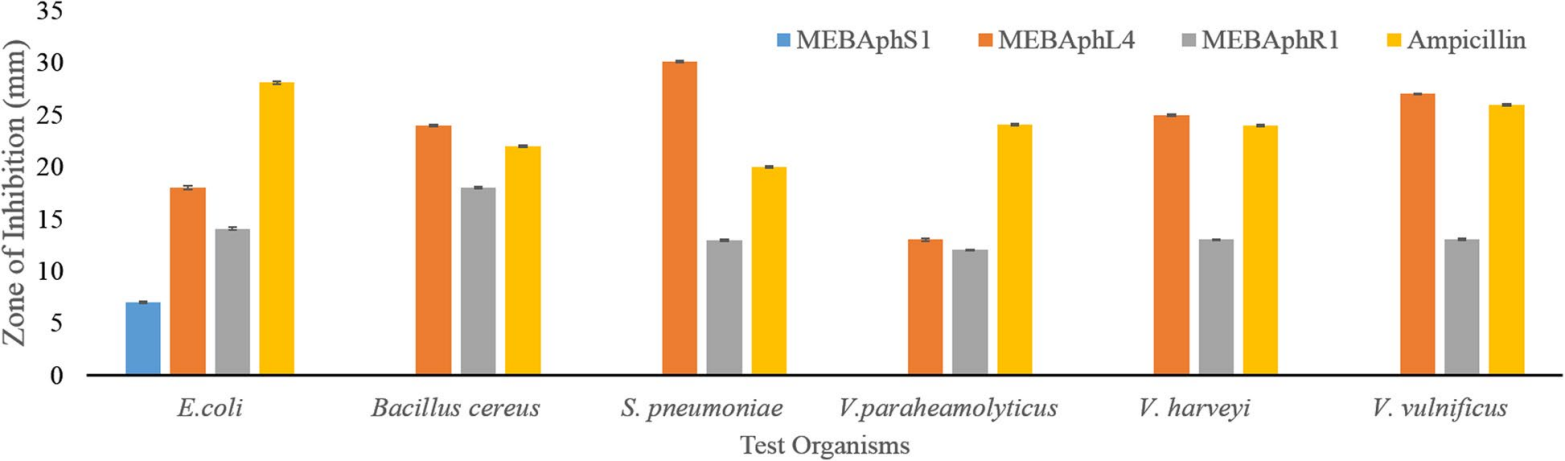
Antibacterial activity of the isolated bacterial endophyte against the selected test organisms *Escherichia coli*, *Bacillus cereus, Streptococcus pneumoniae*, *Vibrio parahaemolyticus*, *Vibrio harveyi*, and *Vibrio vulnificus*

The selected endophytic bacteria were studied further to investigate their in vitro inhibitory activity against *E*. *coli*, *B*. *cereus*, *S*. *pneumoniae*, *V*. *harveyi*,* V*. *parahaemolyticus*, and* V*. *vulnificus.* The isolates *B*. *amyloliquefaciens* and *B*. *subtilis* showed broad-spectrum antibacterial activity against all the six tested organisms. The highest antibacterial activity was found to be by the strain *B*. *amyloliquefaciens* against *S*. *pneumoniae* and the second strongest by *B*. *amyloliquefaciens* against *V*. *vulnificus*. *K*. *pneumoniae* showed the least antibacterial activity against the selected test organisms. Ampicillin was used as a positive control (Fig. 3).

**Fig. 4:**
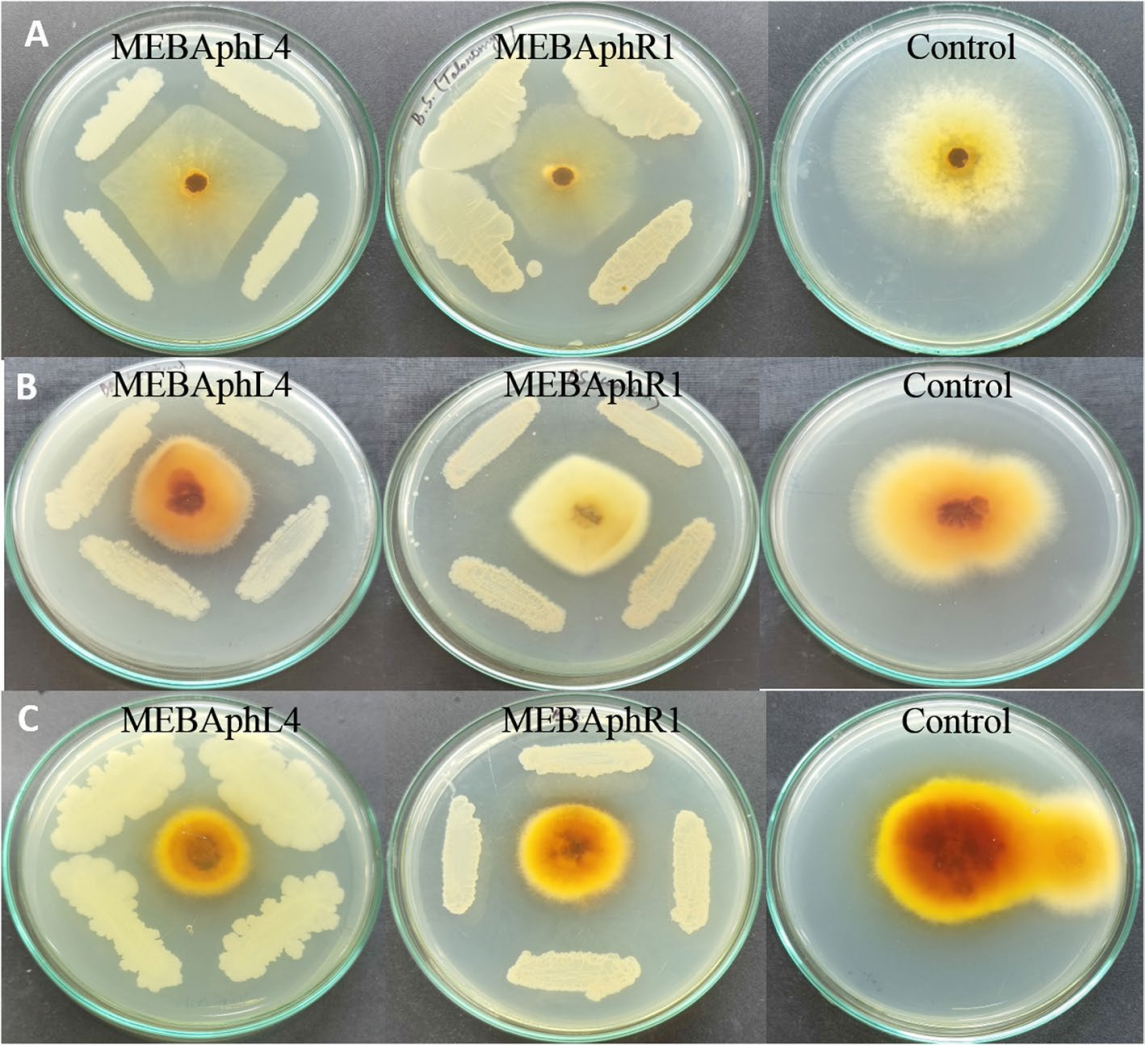
In vitro evaluation of antagonistic activity of bacterial endophyte *Bacillus amyloliquefaciens* MEBAphL4 and *Bacillus subtilis* MEBAphR1 against the fungal pathogen **A ***Talaromyces amistolkiae*, **B ***Fusarium solani*, and **C ***Cladosporium tenuissimum*

The antifungal activity results showed that the fungal strains *F. solani*, *T*. *amestolkiae*, and *C*. *tenuissimum* could not grow normally after 3 days of incubation with antagonistic bacterial endophytic strains *B*. *amyloliquefaciens* and* B*. *subtilis*. The shrinkage and distortion of *T*. *amestolkiae* and *C*. *tenuissimum* and *F*. *solani* hyphae were observed when the fungus was inoculated with *B*. *amyloliquefaciens* and* B*. *subtilis*. The endophytic strains thus were able to control the growth of the fungal mycelium after 3 days of incubation (Fig. 4). The control hyphae were normal and healthy. Additionally, the percentage of inhibition by the *Bacillus* spp. against the tested fungus varied from 37 to 71%. The *B*. *amyloliquefaciens* strain MEBAphL4 and *B*. *subtilis* strain MEBAphR1 showed the largest percent inhibition against *C*. *tenuissimum* (71% and 57%), *T*. *amestolkiae* (60% and 64%), and *F*. *solani* (43% and 37%), respectively. Thus, we hypothesize that the endophytic strains *B*. *amyloliquefaciens* and* B*. *subtilis* secrete some antifungal compounds that may be restricting the growth of fungal phytopathogen. *K*. *pneumoniae* did not show any antifungal activity against the selected fungal strains.

### Characterization of IAA production using GCMS

**Fig. 5:**
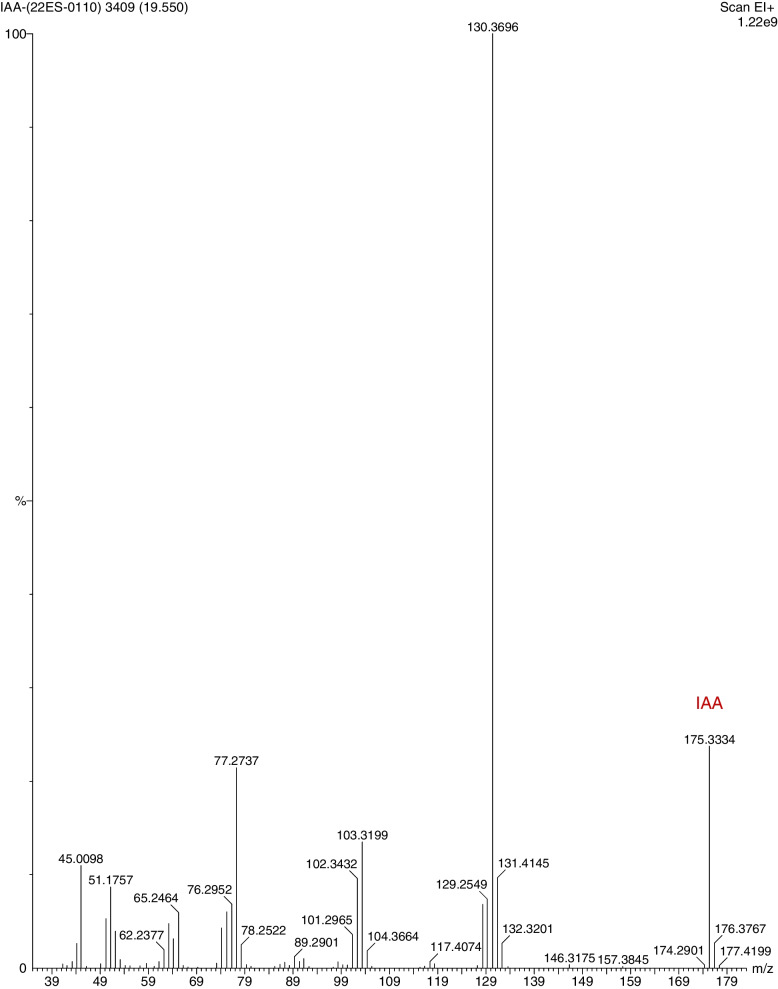
GCMS analysis for IAA in the ethyl acetate extract of endophytic *Klebsiella pneumoniae* MEBAphS1 from *Alternanthera philoxeroides*

The GCMS analysis of the ethyl acetate IAA extract of *K*. *pneumoniae* showed the presence of Indole-3-acetic acid at molecular weight 175.334 g/mol and at retention time 19.55 min, thereby confirming the presence of IAA upon comparison with the reference standard. The base peak detected at molecular weight 130.369 g/mol corresponds to Indolizine-3-methyl- and other ionic peaks at 128.3492 g/mol, 120.4048 g/mol, 105.3431 g/mol, 102.3432 g/mol, 91.3151 g/mol, 77.2737 g/mol corresponds to azulene, benzene-1 ethyl-2 methyl, O-xylene, 3,5 octadiyne, acetic acid,1-methylethyl ester, propanoic acid, 2-methyl- (Fig. 5).

### Optimisation of IAA production by response surface methodology

The optimal levels of factors for the production of IAA by *K*. *pneumoniae* were determined using central composite design containing 30 experiments. The interaction between the independent variables and the response for each experiment were determined based on the quadratic model (Table 7). The fitness of the model was determined by the coefficient of determination (*R*^2^). The value of *R*^2^ more than 0.9 has been considered as a very high correlation between the experimental and predicted values of the postulated model. The *R*^2^ in this model was found to be 0.9701 which shows that 97.01% of the variation that occurred in response could be explained by the model, and the remaining 2.99% could not be explained by the model. The very low *p* value (*p* < 0.0001) suggests that the model fits the experimental data very well and the model terms are significant (Table 8). Regression coefficient and *t* value are inversely proportional to the *p* value, and which is significant from the values of CCD experiments (Table 9). The relationship between the variables for IAA production is explained by substituting the coefficient values from Table 9 into Eq. 1.

**Table 7:** Experimental design of factors for IAA optimisation by *Klebsiella pneumoniae* MEBAphS1, an endophyte isolated from *Alternanthera philoxeroides*

Run	A: Time (days)	B: pH	C: Tryptophan conc. (µg/ml)	Response: IAA conc. (µg/ml)
1	10	6.5	1.05	195.417
2	0	4	0.1	0.672
3	0	4	0.1	0.672
4	26.8179	6.5	1.05	143.132
5	26.8279	6.5	1.05	143.133
6	10	6.5	1.05	195.417
7	20	4	0.1	16.231
8	20	4	0.1	16.231
9	10	10.7045	1.05	120.781
10	10	10.7045	1.05	120.782
11	20	9	2	89.721
12	20	9	2	89.724
13	0	9	0.1	2.72
14	0	9	0.1	2.72
15	0	4	2	2.6395
16	0	4	2	2.6394
17	20	4	2	36.172
18	20	4	2	36.172
19	10	6.5	1.05	195.417
20	10	2.29552	1.05	8.124
21	10	2.29552	1.05	8.135
22	20	9	0.1	92.5455
23	20	9	0.1	92.5455
24	10	6.5	1.05	195.417
25	0	9	2	5.32
26	0	9	2	5.42
27	10	6.5	1.05	195.417
28	10	6.5	1.05	195.417
29	10	6.5	2.6477	43.081
30	10	6.5	2.6477	43.081

**Table 8:** Analysis of Variance (ANOVA) for response surface quadratic model for the optimisation of IAA production by endophyte *Klebsiella pneumoniae* MEBAphS1

Sum of squares	Degree of freedom	Mean square	*f* value	*p* value^*^	*R*^*2*^	Adjusted *R*^*2*^
1.591E + 05	9	17680.03	72.02	< 0.0001	0.9701	0.9566

^*^Statistically significant at *p* < 0.05 probability

**Table 9:** Regression coefficient values from central composite design experiments for IAA optimisation by endophyte *Klebsiella pneumoniae* MEBAphS1

Factors	Coefficient (b_0_-b_9_ of Eq. 1)	Standard error	*t* value	*p* value
Intercept	196.48	6.39	30.74	< 0.0001
A	31.83	3.75	8.488	< 0.0001
B	23.73	3	7.91	< 0.0001
C	6.64	3.75	1.77	0.0919
AB	15.64	3.92	3.989	0.0007
AC	1.56	3.92	0.397	0.6942
BC	− 2.76	3.92	− 0.704	0.4891
A^2^	− 44.38	4.32	− 10.273	< 0.0001
B^2^	− 49.98	3.41	− 14.656	< 0.0001
C^2^	− 64.78	4.32	− 14.995	< 0.0001

$$Y=196.48+31.83 \times \mathrm{ A }+ 23.73 \times \mathrm{ B }+ 6.64 \times \mathrm{ C }+ 15.64 \times \mathrm{ AB }+ 1.56 \times \mathrm{ AC }- 2.76 \times \mathrm{ BC }- 44.38 \times {\mathrm{A}}^{2} - 49.98 \times {\mathrm{B}}^{2} - 64.78 \times {\mathrm{C}}^{2}$$

**Fig. 6:**
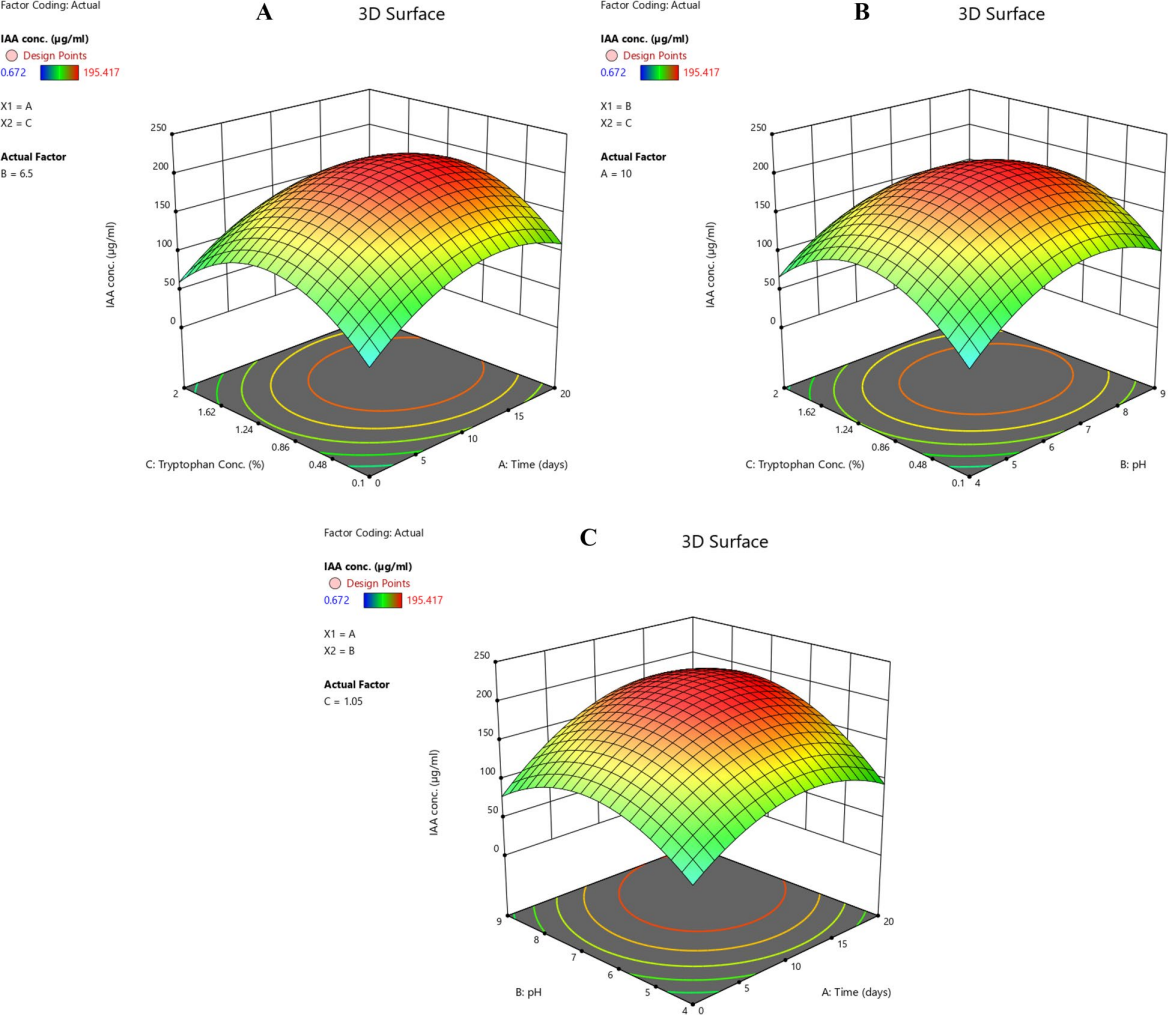
**A** Response surface plot of the effect of interaction between tryptophan concentration and incubation time on IAA production by endophytic *Klebsiella pneumoniae* MEBAphS1 from *Alternanthera philoxeroides*. **B** Response surface plot of the effect of interaction between tryptophan concentration and pH on IAA production by endophytic *Klebsiella pneumoniae* MEBAphS1 from *Alternanthera philoxeroides*. **C** Response surface plot of the effect of interaction between pH and incubation time on IAA production by endophytic *Klebsiella pneumoniae* MEBAphS1 from *Alternanthera philoxeroides*

The linear effect of incubation time and pH with *p* < 0.0001 were more significant than tryptophan concentration, while the quadratic effect of all the three factors were significant with *p* < 0.0001. The interaction between incubation time and pH was more significant with *p* < 0.0007 than other interactions. The interaction between two different variables on IAA production was studied using 3D response surface plots while keeping the third variable at a constant level (Fig. 6A–C). Initially, IAA production increased significantly with increasing days of incubation, pH, and tryptophan concentration but after reaching an optimal level at the 10th day of incubation, pH 6.5 and 1.05% of tryptophan concentration, the IAA production has slightly decreased. Thus, the parameters for optimum IAA production by *K*. *pneumoniae* were 10 days of incubation, pH 6.5, and tryptophan concentration of 1.05%.

### Pot trial experiment

**Fig. 7:**
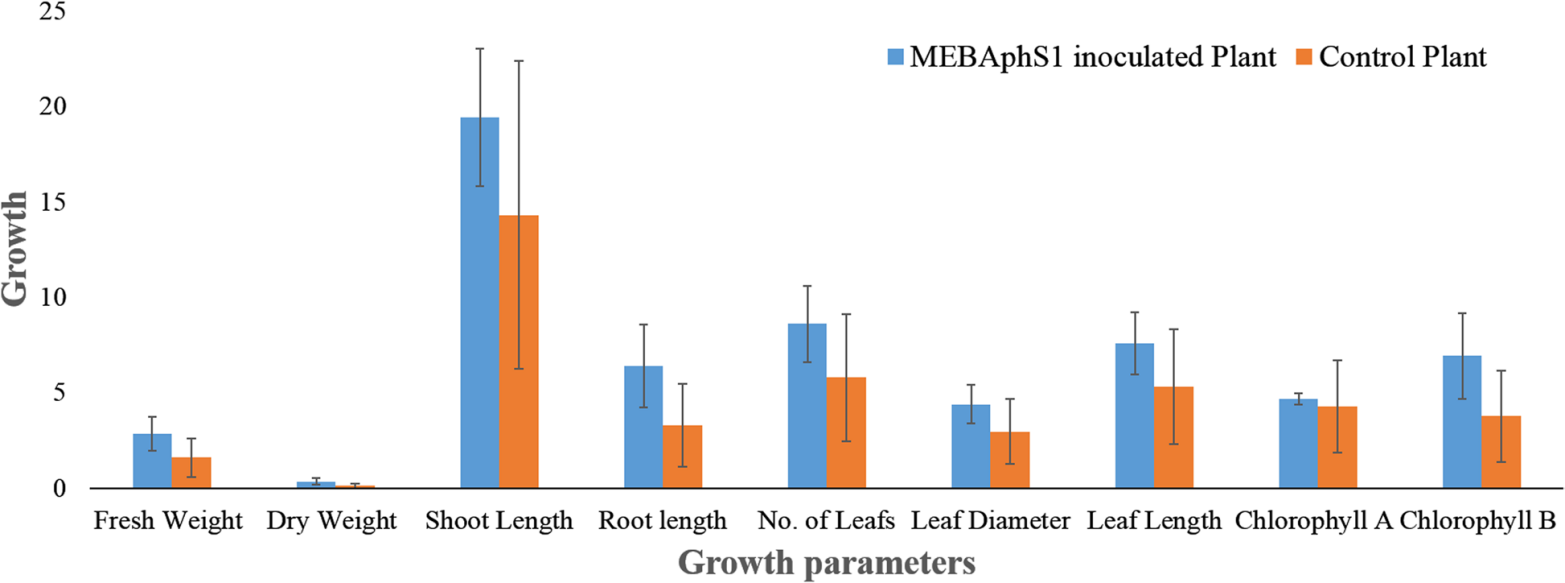
Comparison of growth parameters of endophyte *Klebsiella pneumoniae* MEBAphS1 treated and control *Vigna unguiculata* plant

**Fig. 8:**
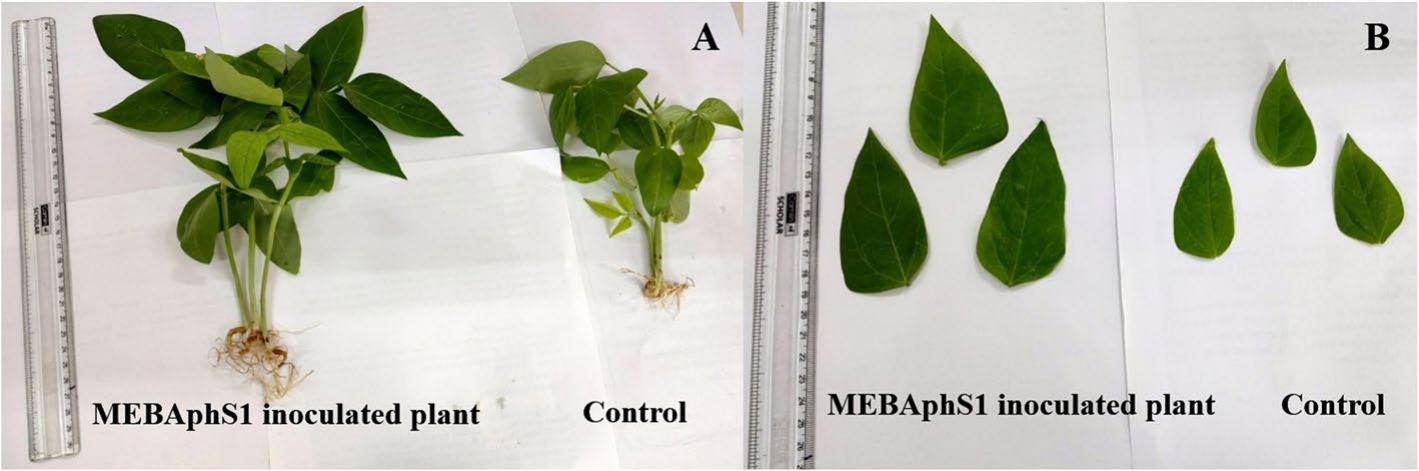
Phenotypic expression of *Vigna unguiculata* plant inoculated with endophytic bacteria *Klebsiella pneumoniae* MEBAphS1 showed **A** increased plant height, and **B** increased leaf length and diameter in *Klebsiella pneumoniae* inoculated plant compared to control plant

Seeds of *V*. *unguiculata* plant inoculated with endophytic bacteria *K*. *pneumoniae* MEBAphS1 have shown a dramatic increase in overall plant growth as compared to control non-treated plant (Figs. 7 and 8). All the studied growth parameters were significantly (*p* ≤ 0.05) higher in inoculated plants than in control plants. Fresh weight and dry weight in MEBAphS1 inoculated *V*. *unguiculata* plants were significantly higher (2.86 ± 0.21; *p* ≤ 0.05 and 0.352 ± 0.08; *p* ≤ 0.05 respectively) than the control plants. The treated plants had shown increased shoot length (19.4 ± 1.24;* p* ≤ 0.05) and root length (6.4 ± 1.01; *p* ≤ 0.05) in comparison to non-treated plants. Similarly, different leaf parameters like leaf number, leaf diameter, and length were found to be significantly on the higher side for inoculated plants in comparison with the non-inoculated plants. Chlorophyll B content showed a significant increase in the inoculated plants compared to the control (Table 10).

**Table 10:** Plant growth promotion in *Vigna unguiculata* plant inoculated with endophyte *Klebsiella pneumoniae* MEBAphS1. Means are averages ± SD (*n* = 5). Student’s *t* test at *p* ≤ 0.05

Treatment	Fresh weight (g)	Dry weight (g)	Shoot length (cm)	Root length (cm)	No. of leafs	Leaf diameter (cm)	Leaf length (cm)	Chlorophyll A	Chlorophyll B
*K. pneumoniae* inoculated plant	2.86 ± 0.21	0.352 ± 0.08	19.4 ± 1.24	6.4 ± 1.01	8.6 ± 1.20	4.4 ± 0.48	7.6 ± 0.58	4.69 ± 0.63	6.93 ± 0.45
Control Plant	1.6 ± 0.20	0.126 ± 0.02	14.3 ± 2.74	3.3 ± 0.60	5.8 ± 1.16	2.96 ± 0.32	5.3 ± 0.40	4.28 ± 0.33	3.77 ± 0.18

## Discussion

Endophytic associations would have survived the test of time mostly due to the benefits conferred to both parties. Endophytic bacteria in plants provide and receive help in multifaceted ways. In the present study, we have reported the presence of gram-negative and gram-positive bacterial endophytes from different parts of the aquatic plant *A*. *philoxeroides* from Madiwala Lake in Bangalore. The identity of the prominent strains was confirmed after phylogenetic analysis as *K*. *pneumoniae*, *B*. *amyloliquefaciens*, and* B*. *subtilis*. Different tests have confirmed that all these three isolates have good PGP properties and stress tolerance abilities and can also act as potential biocontrol agents.

In a recent study, three endophytes belonging to *Pseudomonas* genus isolated from sorghum were found to be able to tolerate up to 7.2% NaCl concentration, which is much higher than the salt tolerance level of the plant alone [48]. Another study reported that seedlings of *Salicornia bigelovii* upon inoculation with PGP rhizobacteria *K*. *pneumoniae* at a high salt concentration of 0.25 M (1.45%) exhibited increased plant growth and physiological activity under in vitro conditions [49]. Salt-tolerant PGP rhizobacteria *K*. *pneumoniae* have been reported to tolerate NaCl concentrations up to 20% and also promote plant growth in oats seedlings [50]. Liu et al. (2016) have reported that PGP rhizobacterium *Klebsiella* spp. D5A was able to tolerate up to 12% NaCl concentration and has also revealed the presence of salt-tolerant genes such as *betA* and *betB* [51]. Our present study has shown that the endophytic strain *K*. *pneumoniae* can tolerate up to 10% NaCl concentration and also has good PGP traits. Thus, the potential of this isolate can be harnessed in saline soils to enhance agricultural productivity.

Kumar et al. (2016) have demonstrated that the three bacterial endophytes of *Bacillus* spp. isolated from a *Curcuma longa* L. with PGP traits and antimicrobial activity were able to tolerate a maximum of 8% NaCl concentration [52]. Sharma et al. (2021) have isolated bacterial isolates *B*. *amyloliquefaciens*, *B*. *paramycoides*, and* B*. *pumilus* from salt-polluted soil with PGP ability and able to tolerate up to 10% of NaCl concentration [23]. Bacterial endophyte *B*. *amyloliquefaciens* RWL-1, isolated from rice seeds, has been reported to produce plant hormone abscisic acid which increases plant resistance to salt stress. This endophyte was able to tolerate salt stress of 120 mM and 250 mM NaCl concentration [53]. In our present study, two of the bacterial endophytes *B*. *amyloliquefaciens* and *B*. *subtilis* were found to tolerate up to 15% NaCl concentration. Much of the earlier works have shown a salt tolerance level of up to 10% among bacterial endophytes. The two strains along with tolerance to higher salt concentrations also showed good PGP capabilities and biocontrol ability. Their ability to confer salt tolerance in plants could be of great potential in agriculture, to increase crop yield under salinity stress.

Extracellular enzymes in endophytic bacteria have been found to help in the degradation of the cell wall of various pathogens, thereby acting as a biocontrol agent [54]. Castro et al. (2014) reported many species of *Bacillus* from plants in the Brazilian mangrove ecosystem with extracellular enzyme activity with industrial implications, like amylase, lipase, esterase, protease, and cellulase [55]. Bhutani et al. (2021) have reported the presence of hydrolytic enzymes such as amylase, cellulase, lipase, protease, and pectinase among different *Bacillus* spp. isolated from *V*. *radiata* and *Cajanus cajan*, which can lyse the cell wall, loosen the plant cell contacts, and enter the plant cells [56]. In the present study, we confirmed that *B*. *amyloliquefaciens* and *B*. *subtilis* possess extracellular enzyme activity for all the four tested enzymes such as amylase, cellulase, lipase, and protease, revealing their potential of improving growth by indirect mechanisms, thereby acting as a potential biocontrol agent.

IAA is an important PGP hormone produced by many bacteria through tryptophan-dependent and independent pathways. The phytohormone IAA plays an essential role in plants by promoting cell division, elongation, lateral and adventitious root development, fruit development, and senescence, and also by acting as a signaling molecule [57]. Bacterial IAA loosens the cell wall and increases the root length and surface area, thereby enhancing the uptake of nutrients by the plants. Many plant growth-promoting bacteria (PGPB) strains with potent IAA production potential have been reported among *Bacillus* spp., *Klebsiella* spp., *Azotobacter* spp., *Agrobacterium* spp., *Pseudomonas* spp., *Streptomyces* spp., *Burkholderia* spp. [6]. In a recent study [58], *K*. *pneumonia* strain M6 isolated from mango rhizosphere had shown IAA production of 35.53 ± 0.2 µg/ml in tryptic soy broth (TSB) media amended with 0.5% tryptophan. Jasim et al. (2013) have reported PGP endophytic *Klebsiella* spp. and *Enterobacter* spp. isolated from *Piper nigrum* with IAA biosynthetic potential [36]. Six *K*. *pneumoniae* strains isolated from the rhizosphere of wheat showed in vitro IAA production with a maximum of 27.5 µg/ml shown by K8 strain [59]. Bharadwaj et al. (2017) have reported IAA production of 45.32 ± 2.46 µg/ml by *K*. *pneumoniae* VRE36 strain after 96 h of incubation at 37 °C [60]. Pavlova et al. (2017) have isolated PGP endophytic bacteria *Klebsiella* spp. from orchids with the highest IAA production of 85.5 µg/ml under nitrogen-limiting conditions. *Klebsiella* spp. have been reported to possess efficient colonization activity and growth-promoting activity upon tryptophan addition [61]. Our study reports very high IAA production by *K*. *pneumoniae*, 48.75 ± 3 µg/ml after 72 h of incubation at 37 °C. Further, the IAA production was enhanced after the optimization process.

In a recent study by Kang et al. (2020), endophytic bacteria *K*. *pneumoniae* YNA12 was isolated from evening primrose and screened for various PGP properties and bio-herbicidal activity. *Klebsiella* strain YNA12 exhibited significant ammonia-producing potential with a reported concentration of 7.75 ± 1.0 µg/ml [62]. In our study, we have detected 3.51 ± 0.15 µg/ml of ammonia production by *B*. *amyloliquefaciens* and 2.92 ± 0.05 µg/ml by *K*. *pneumoniae*. Sharma et al. (2021) have demonstrated that salt-tolerant PGPB, *B*. *amyloliquefaciens* has shown ammonia production of 6.2 µg/ml [23].

Nitrogen, phosphorus, and potassium are essential to plant growth-limiting nutrients. Although phosphorus and potassium are present in abundant amounts in soil, they remain bound to other minerals and are mostly insoluble, and therefore remain unavailable to plants [63]. Phosphorus is one of the essential macronutrients for plant growth and development. The available precipitated form of phosphorus cannot be used by plants; therefore, phosphate solubilizing microbes are of great importance as they make available the phosphorus in a readily accessible form to the plants [64]. According to Gupta et al. (2021), PGP *K*. *pneumoniae* PNE1 has shown a phosphate solubilization index of 4.16 and has solubilized 177.50 µg/ml [39]. Bacterial endophytic strain 2106 isolated from maize which showed high similarity to *Bacillus* spp. was able to solubilize phosphate up to 191.46 mg/L when inoculated into NBRIP liquid medium and quantified by ammonium molybdate colorimetric method [65]. In our study, the phosphate solubilization index was 1.6, 1.4, and 1.3 for *K*. *pneumoniae*, *B*. *amyloliquefaciens*, and *B*. *subtilis* strains respectively. *K*. *pneumoniae* was able to solubilize 38.625 ppm of phosphate. In our study, all three isolated endophytic bacteria *K*. *pneumoniae*, *B*. *amyloliquefaciens*, and *B*. *subtilis* also have shown luxuriant growth in Jensen’s media indicating their nitrogen-fixing ability. Several scientific reports have suggested that *K*. *pneumoniae* grows in nitrogen-free media by fixing atmospheric nitrogen [39, 66]. Mowafy et al. (2021) have reported that *Bacillus* B2L2 was able to grow on Jensen’s media and therefore can fix nitrogen [67]. In the present study, *K*. *pneumoniae* showed a good potassium solubilization zone in Aleksandrow media and quantitative estimation revealed inorganic potassium solubilization to be 2.13 ± 0.07 ppm after 192 h of incubation. The concentration of phosphate solubilization by the isolates largely depends upon the type of bacterial isolate, phosphate sources, growth of the organism in culture, and environmental conditions. In a recent study by Gupta et al. (2021), *K*. *pneumoniae* strain PNE1 has shown a potassium solubilization index (KSI) of 3.6 in Aleksandrow media, and quantitative data revealed maximum potassium solubilization of 29.94 mg/L on 21st day of incubation [39]. Wang et al. (2020) have reported KSI of 1.57 by PGPB *K*. *pneumoniae* K6 and potassium solubilization to be 38.55 ± 2.82 mg/L. Similarly, for different *K*. *variicola* strains, KSI ranged from 1.39 to 2.78, and potassium solubilization by *K*. *variicola* K5 was 64.16 ± 2.19 mg/L [68].

Cui et al. (2021) reported the endophytic bacterium *B*. *amyloliquefaciens* to show antibacterial activity against *Streptomyces griseoplanus* which causes potato scabs. Six diverse antibacterial compounds were detected after PCR analysis [69]. Iturin and surfactin-like compounds were reported from endophytic *B*. *amyloliquefaciens* showing inimical activity against *Streptococcus*, *Staphylococcus*, and *Cryptococcus* strains [70]. Endophytic *B*. *subtilis* strain 1-L-29 has been found to show antimicrobial activity against a wide range of bacterial and fungal pathogens along with PGP properties [71]. In a recent study by Soliman et al. (2022), endophytic *B*. *amyloliquefaciens* MZ945930 and *B*. *velezensis* MZ945929 showed antifungal activity against *Alternaria* spp. and *Helminthosporium* spp. [72]. The endophytic *B*. *subtilis* strain isolated from the maize plant exhibited good PGP attributes and biocontrol activity against the fungus *Botrytis cinerea* [73]. Mohamad et al. (2020) reported that the *Bacillus* genus showed high antagonistic activity against the tested fungal strains and the percentage of inhibition ranges from 40 to 77% [42]. In our present study, *B*. *amyloliquefaciens* and *B*. *subtilis* both showed a broad range of antibacterial and antifungal activity against the selected bacterial and fungal pathogenic strains. Both the strains showed salt tolerance to 15% of NaCl concentration, and had high levels of ammonia production, nitrogen-fixing ability, phosphate solubilization, and ACC deaminase activity, thereby helping plants in both direct and indirect ways of PGP and acting as promising candidates for biofertilizers and biocontrol agents.

The central composite design of response surface methodology (RSM) used for the optimization of IAA production has helped us to evaluate the interaction between different variables. Optimization of IAA production for rhizospheric bacteria *Klebsiella* SN 1.1 recorded the highest IAA production of 291.97 ± 0.19 µg/ml at 0.2% tryptophan after 9 days of incubation [74]. Lebrazi et al. (2020) have reported an optimal IAA production of 166 µg/ml by rhizospheric bacteria at pH 6.5, temperature 36 °C, tryptophan and NaCl concentration of 1 g/L and 0.1 g/L with an incubation time of 1 day [45]. The deployment of the experimental design has allowed us to get a maximal yield of IAA production by *K*. *pneumoniae*—195.417 µg/ml—with 10 days of incubation time, pH 6.5, and 1.05% of tryptophan concentration. The obtained pH 6.5 by the experimental design is similar to those reported by Lebrazi and coworkers [45]. The obtained responses from the experiment had also shown a close match with the predicted responses and thus validated the experimental model.

Ours is the first report of the presence of *Klebsiella* spp. as an endophyte with IAA production in *A*. *philoxeroides*. With a view to checking the effectiveness as a microbial inoculant, *K*. *pneumoniae* was used for seed treatment of cowpeas. In our present study, the seeds of *V*. *unguiculata* treated with *K*. *pneumoniae* showed increased shoot and root length, number of leaves, leaf diameter and length, fresh and dry weight, and chlorophyll content as compared to a control plant. The results were found to be statistically significant (*p* ≤ 0.05) in comparison to control plants. *K*. *pneumoniae* showed higher IAA production, phosphate solubilization, potassium solubilization, and nitrogen-fixing ability which might have influenced the germination capacity and growth of the plant. Jasim et al. (2013) have also reported the inoculation of culture supernatant of endophytic bacterial strains *Klebsiella* spp. and *Enterobacter* spp. isolated from *P*. *nigrum* have increased the shoot and root length among seedlings of *V*. *radiata* as compared to the control [36]. The study by Dey et al. (2019) revealed that endophytic *K*. *pneumoniae* HR1 isolated from *V*. *mungo* L. have significantly increased the germination capacity, root and shoot length, dry weight of root and shoot in treated and control plants and *Macrophomina phaseolina* infected plant, thereby making endophytic *K*. *pneumoniae* a potential PGP and biological control agent [75]. More diverse enzymes and metabolites are found to be generally harbored by endophytic fungi [76–78] and bacteria [79] isolated from both normal and stressed environments, many of them possessing excellent PGP properties [80].

Our results suggest that the stem isolate *K*. *pneumoniae* possesses multiple PGP traits that directly help in plant growth, while the leaf isolates *B*. *amyloliquefaciens* and root isolate *B*. *subtilis* has good extracellular enzyme activity and PGP abilities and the broad range of antimicrobial activity help both directly and indirectly in plant growth and as biocontrol agent. Thus, these isolates may be utilized for improving crop yield in sustainable agriculture.

## Conclusion

The use of endophytes to improve plant growth and protect crops from pathogens can be the most sustainable and cost-effective approach in agriculture as it is in tune with the natural settings of the environment. In the present study, the *A*. *philoxeroides* stem isolate, *K*. *pneumoniae* showed multiple PGP traits that resulted in improved germination and vegetative growth of *V*. *unguiculata* plant. *B*. *amyloliquefaciens* and* B*. *subtilis* showed good extracellular enzyme production and antimicrobial activity along with plant growth promotion. Given the multiple PGP attributes of these strains, these can be commercially utilized in agriculture instead of chemical fertilizers. Thus, a consortium of these isolates could be further used as bioinoculants for improving plant growth in crop plants after validation by field trials, to increase crop yield under salinity stress. These kinds of studies align with the United Nations SDGs (Sustainable Development Goals) aiming at finding means to improve crop productivity without harming the environment much, as indeed it is the need of the hour.

### Supplementary Information

**Additional file 1: Fig. S1.** (A) Phylogenetic analysis of 16S rRNA sequences of *Klebsiella pneumoniae* MEBAphS1 from *Alternanthera philoxeroides*, reflecting the relationship with the ITS sequences of closely related *Klebsiella* strains retrieved from the NCBI GenBank database. The phylogenetic tree was constructed using MEGA 10. **Fig. S1** (B) Phylogenetic analysis of 16S rRNA sequences of *Bacillus amyloliquefaciens* MEBAphL4 from *A. philoxeroides*, reflecting the relationship with the ITS sequences of closely related *Bacillus* strains retrieved from the NCBI GenBank database. The phylogenetic tree was constructed using MEGA 10. **Fig. S1.** (C) Phylogenetic analysis of 16S rRNA sequences of *Bacillus subtilis* MEBAphR1 from *Alternanthera philoxeroides*, reflecting the relationship with the ITS sequences of closely related *Bacillus* strains retrieved from the NCBI GenBank database. The phylogenetic tree was constructed using MEGA 10.

## Data Availability

All data generated or analyzed during this study are included in this published article (and its supplementary information files).

## Abbreviations

PGP: Plant growth promotion
PGPEB: Plant growth promoting endophytic bacteria
IAA: Indoleacetic acid
RSM: Response surface methodology
GCMS: Gas chromatography mass spectrometry
PGPB: Plant growth promoting bacteria
NA: Nutrient agar
ACC: 1-Aminocyclopropane-1-carboxylic acid
HT-PGPB: Halo-tolerant plant growth promoting bacteria
ROS: Reactive oxygen species
LB: Luria-Bertani
PCR: Polymerase chain reaction
EI: Enzymatic Index
MSM: Mineral salt media
DF: Dowrkin-Foster
HCN: Hydrogen cyanide
PSI: Phosphate Solubilization Index
AAS: Absorption spectrophotometer
SDA: Sabouraud dextrose agar
CCD: Central composite design
SD: Standard deviation
ANOVA: Analysis of variance
KSI: Potassium Solubilization Index
TSB: Tryptic soy broth
SDGs: Sustainable development goals
