# Supplementary figures and images for: Endophytic bacteria Klebsiella spp. and Bacillus spp. from Alternanthera philoxeroides in Madiwala Lake exhibit additive plant growth-promoting and biocontrol activities
Source: J Genet Eng Biotechnol. 2023 Nov 30;21:153. doi: 10.1186/s43141-023-00620-8 (PMC10686955; doi:10.1186/s43141-023-00620-8)

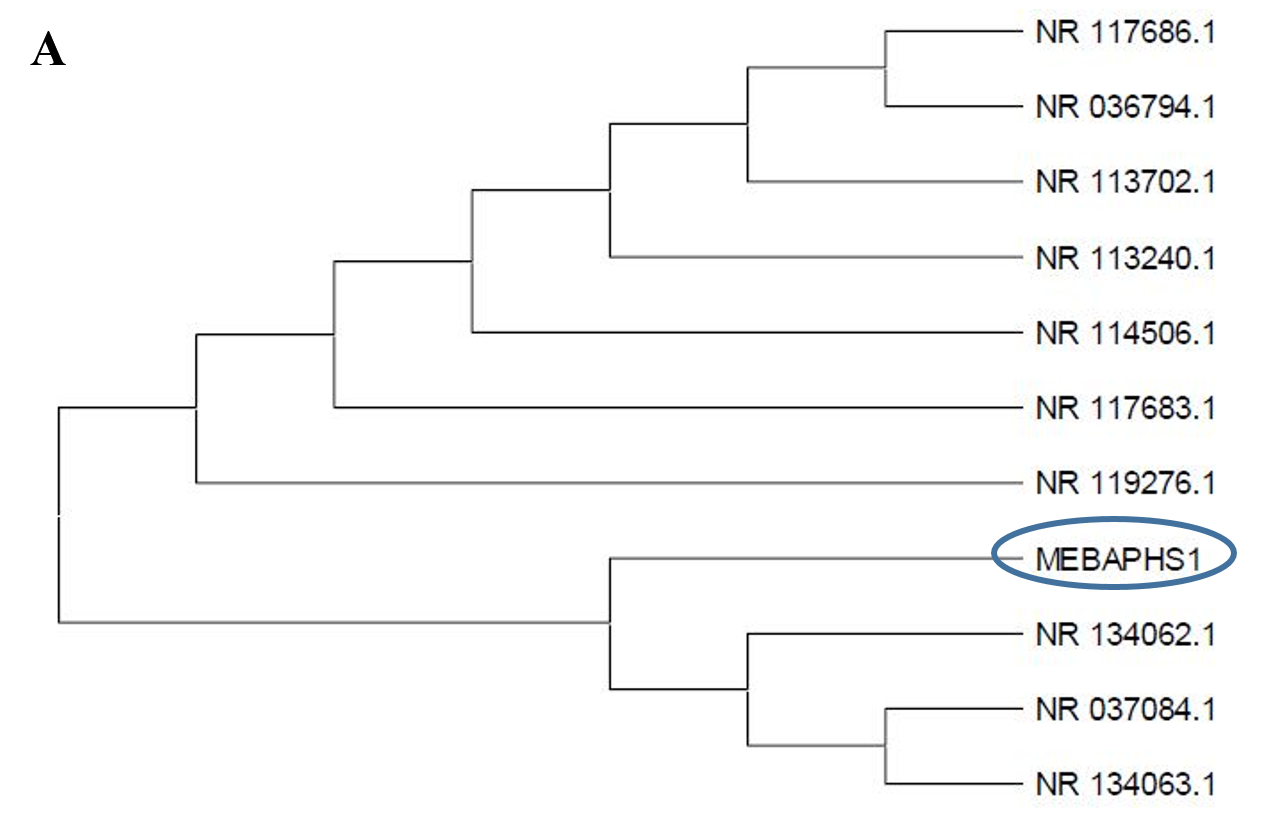

Supplement: Supplementary file 1 — Additional file 1: Fig. S1. (A) Phylogenetic analysis of 16S rRNA sequences of Klebsiella pneumoniae MEBAphS1 from Alternanthera philoxeroides, reflecting the relationship with the ITS sequences of closely related Klebsiella strains retrieved from the NCBI GenBank database. The phylogenetic tree was constructed using MEGA 10. Fig. S1 (B) Phylogenetic analysis of 16S rRNA sequences of Bacillus amyloliquefaciens MEBAphL4 from A. philoxeroides, reflecting the relationship with the ITS sequences of closely related Bacillus strains retrieved from the NCBI GenBank database. The phylogenetic tree was constructed using MEGA 10. Fig. S1. (C) Phylogenetic analysis of 16S rRNA sequences of Bacillus subtilis MEBAphR1 from Alternanthera philoxeroides, reflecting the relationship with the ITS sequences of closely related Bacillus strains retrieved from the NCBI GenBank database. The phylogenetic tree was constructed using MEGA 10. [file 43141_2023_620_MOESM1_ESM.zip › Fig. S1(A)R3.tif]

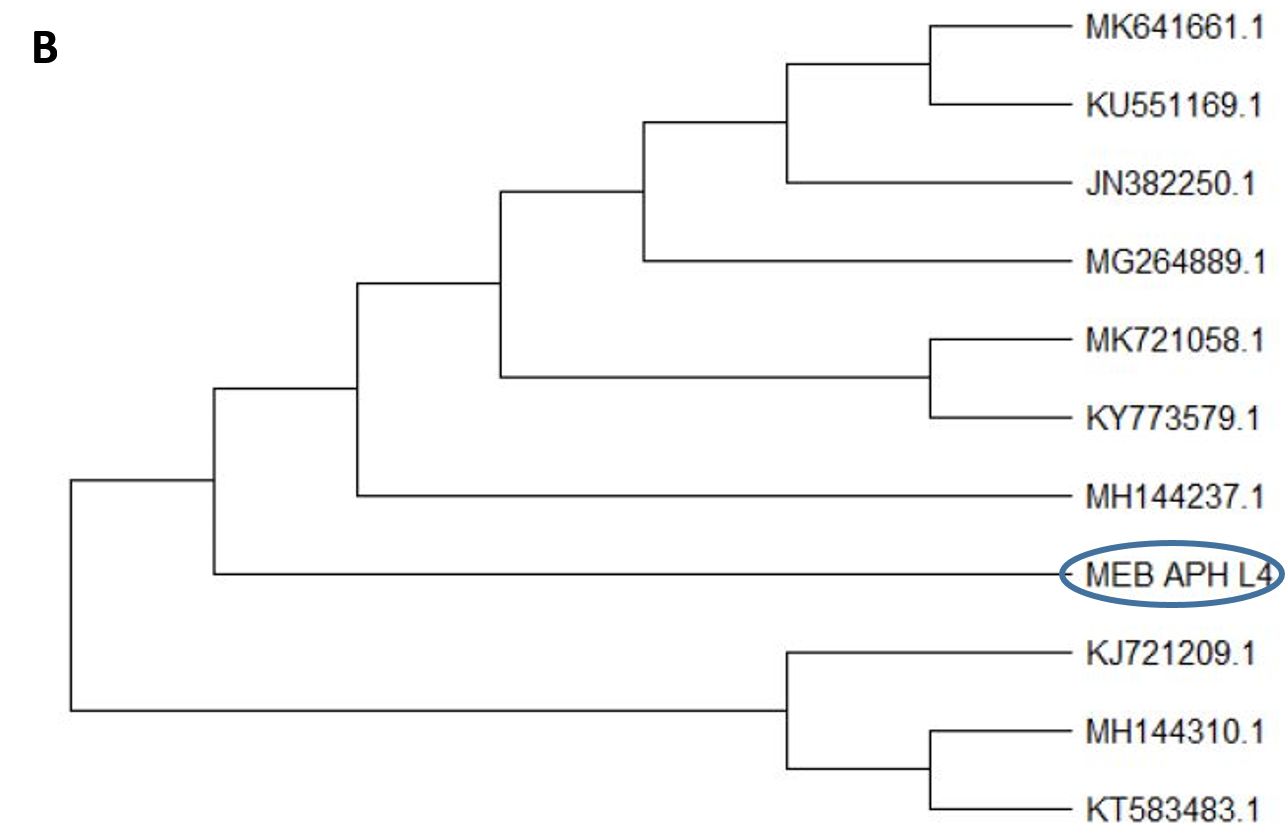

Supplement: Supplementary file 1 — Additional file 1: Fig. S1. (A) Phylogenetic analysis of 16S rRNA sequences of Klebsiella pneumoniae MEBAphS1 from Alternanthera philoxeroides, reflecting the relationship with the ITS sequences of closely related Klebsiella strains retrieved from the NCBI GenBank database. The phylogenetic tree was constructed using MEGA 10. Fig. S1 (B) Phylogenetic analysis of 16S rRNA sequences of Bacillus amyloliquefaciens MEBAphL4 from A. philoxeroides, reflecting the relationship with the ITS sequences of closely related Bacillus strains retrieved from the NCBI GenBank database. The phylogenetic tree was constructed using MEGA 10. Fig. S1. (C) Phylogenetic analysis of 16S rRNA sequences of Bacillus subtilis MEBAphR1 from Alternanthera philoxeroides, reflecting the relationship with the ITS sequences of closely related Bacillus strains retrieved from the NCBI GenBank database. The phylogenetic tree was constructed using MEGA 10. [file 43141_2023_620_MOESM1_ESM.zip › Fig. S1(B)R3.tif]

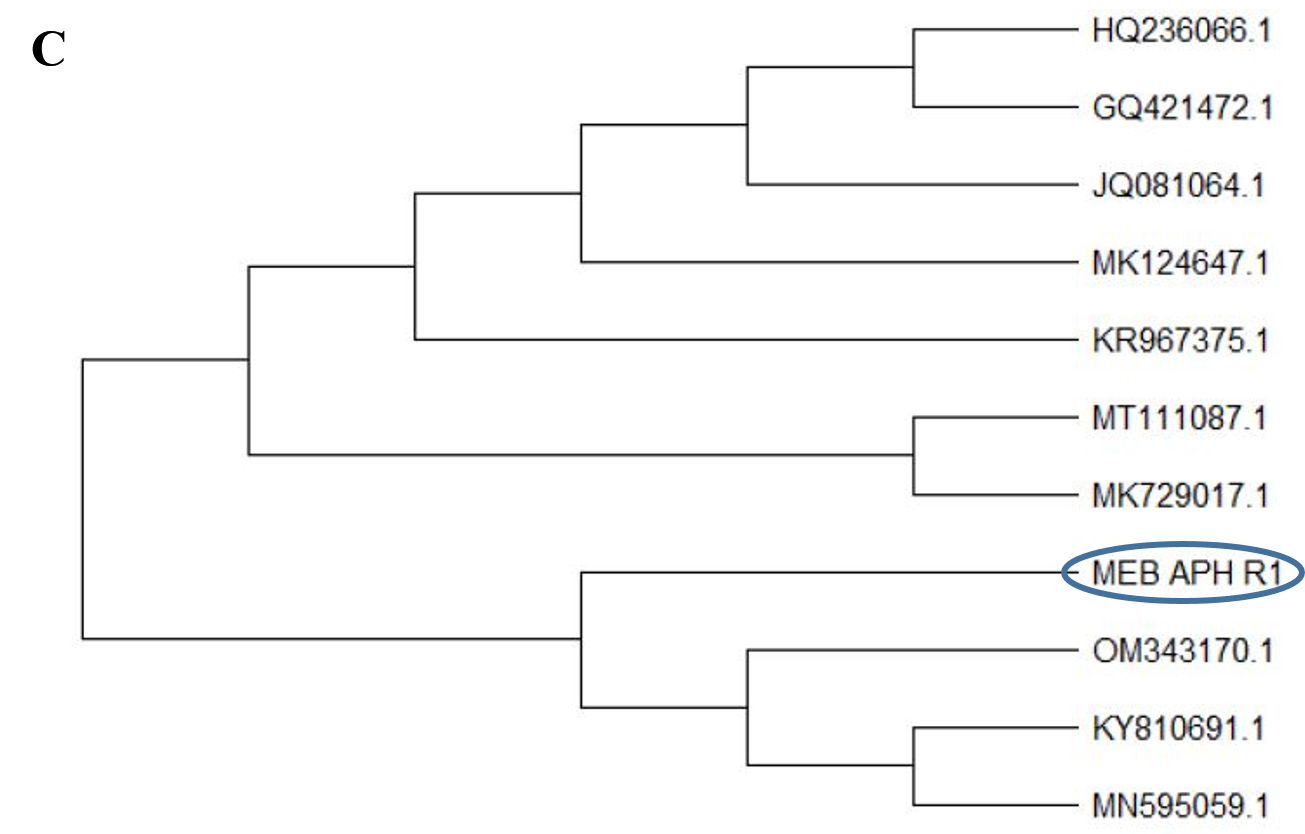

Supplement: Supplementary file 1 — Additional file 1: Fig. S1. (A) Phylogenetic analysis of 16S rRNA sequences of Klebsiella pneumoniae MEBAphS1 from Alternanthera philoxeroides, reflecting the relationship with the ITS sequences of closely related Klebsiella strains retrieved from the NCBI GenBank database. The phylogenetic tree was constructed using MEGA 10. Fig. S1 (B) Phylogenetic analysis of 16S rRNA sequences of Bacillus amyloliquefaciens MEBAphL4 from A. philoxeroides, reflecting the relationship with the ITS sequences of closely related Bacillus strains retrieved from the NCBI GenBank database. The phylogenetic tree was constructed using MEGA 10. Fig. S1. (C) Phylogenetic analysis of 16S rRNA sequences of Bacillus subtilis MEBAphR1 from Alternanthera philoxeroides, reflecting the relationship with the ITS sequences of closely related Bacillus strains retrieved from the NCBI GenBank database. The phylogenetic tree was constructed using MEGA 10. [file 43141_2023_620_MOESM1_ESM.zip › Fig. S1(C)R3.tif]
